# Clinical applications of carbonated beverages: A case report of large gastric phytobezoar complicated by acute pancreatitis

**DOI:** 10.1097/MD.0000000000046536

**Published:** 2025-12-12

**Authors:** Guobiao Luo

**Affiliations:** a Department of Gastroenterology, Guangzhou Red Cross Hospital, Jinan University, Guangzhou, China.

**Keywords:** acute pancreatitis, comprehensive therapy, gastric ulcers, phytobezoar

## Abstract

**Rationale:**

Phytobezoars are foreign bodies that form in the gastrointestinal tract as a result of accumulated ingested substances. Phytobezoars and acute pancreatitis occur concomitantly are rare, presenting diagnostic and therapeutic challenges for clinicians. Endoscopic mechanical lithotripsy with a single-use snare was utilized in this case; the treatment effect was suboptimal, and oral sodium bicarbonate and carbonated beverages are simple and effective non-surgical treatments for phytobezoars.

**Patient concerns:**

A 65-year-old woman was hospitalized at our facility after experiencing abdominal pain for half a day due to eating persimmons. The diagnosis of phytobezoar-induced pancreatitis and gastric ulcers was confirmed by clinical examination. Most clinical symptoms have improved with comprehensive therapeutic strategies.

**Diagnoses:**

The final diagnosis was gastric phytobezoar, acute pancreatitis, and gastric ulcers.

**Interventions:**

Including anti-infection measures, inhibiting gastric acid secretion, safeguarding the gastric mucosa, inhibiting pancreatic enzyme activity, minimally invasive endoscopic procedures, and drinking carbonated beverages (Coca-Cola).

**Outcomes:**

The majority of clinical symptoms improved with comprehensive therapeutic strategies, and a follow-up gastroscope after 4 days showed that the phytobezoar had disappeared. At the 14-day follow-up, the patient felt well and did not experience any symptoms.

**Lessons:**

This case presents ideas and methods for treating phytobezoars in patients with multimorbidity and is intended to serve as a reference for the clinical diagnosis and treatment of similar situations.

## 1. Introduction

Phytobezoars are foreign bodies that form in the gastrointestinal tract as a result of accumulated ingested substances. Upper gastrointestinal symptoms, such as nausea, vomiting, bloating, and abdominal pain, may result from phytobezoars.^[1,2]^ Different types of bezoars have been described in the literature, among which phyto- and trichobezoars are the most frequent. They can be asymptomatic or present with epigastric pain, pressure-induced ulcer bleeding, gastrointestinal perforation, or small-bowel obstruction. Acute pancreatitis is characterized by abdominal pain and elevated pancreatic enzymes. It is an inflammatory disorder affecting the pancreas, which undergoes self-digestion, leading to acute pancreatitis.^[3,4]^ Phytobezoars and acute pancreatitis are rare and present diagnostic and therapeutic challenges for clinicians. The diagnosis and treatment of phytobezoars with multimorbidity have been reported to summarize clinical experience.

## 2. Case presentation

**Figure 1.**
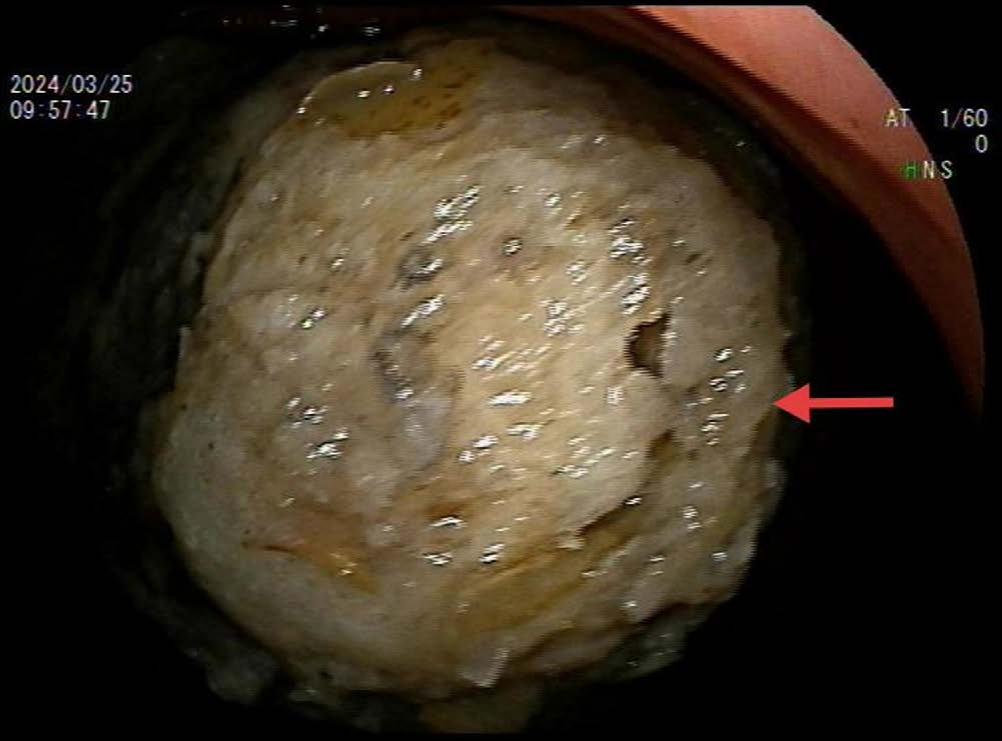
Endoscopy results. The red arrow indicates a large phytobezoar in the gastric body, diameter of the phytobezoar is 5 cm.

**Figure 2.**
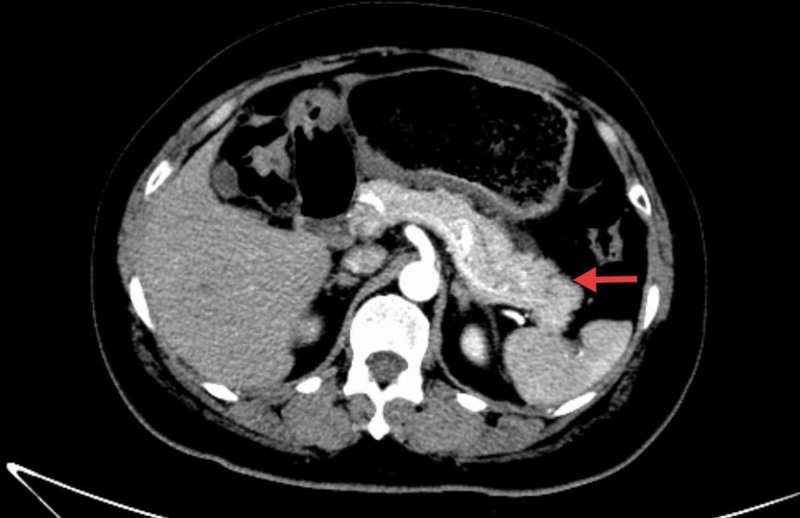
Contrast-enhanced CT of the abdomen. The red arrow indicates mild swelling of the pancreas. CT = computed tomography.

The patient was a 65-year-old female, she was admitted to the hospital on March 20, 2024, for the onset of acute epigastric pain, had eaten persimmons before admission, and had no underlying diseases. Vital signs on admission: body temperature 37°C, blood pressure 141/94 mm Hg, heart rate 71 times/min, respiratory rate 20 times/min, she was conscious and well co-operative, there was no yellow staining on skin and sclera, no abnormal petechia or ecchymosis of the skin was observed, upon auscultation, the lungs were clear, and there were no abnormal heart sounds or murmurs, physical examination on admission revealed that the abdomen was flat and soft, and mild tenderness in the upper abdomen, without rebound tenderness, no palpable mass was identified, her liver and spleen were impalpable, Murphy’s sign and tenderness in the appendix area were negative, the bowel sound was normal, no edema at the lower extremities. Laboratory examinations: blood routine: white blood cell count 13.69 × 10^9^/L, neutrophilic granulocyte ratio 0.772, serum amylase 366 U/L, Gastroscopy: a large phytobezoar in the gastric body, diameter of the phytobezoar is 5 cm (Fig. 1), multiple ulcers in the corners of the stomach, Contrast-enhanced computed tomography of the abdomen revealed thickening and edema of the stomach wall together with peripheral exudate on the side of the wall’s lesser curvature and mild swelling of the pancreas (Fig. 2). Based on the symptoms, auxiliary examinations, and other results, the patient was diagnosed with phytobezoar complicated by acute pancreatitis and multiple ulcers. The patient was administered proton pump inhibitors to inhibit gastric acid production, somatostatin to inhibit pancreatic enzyme secretion, and antibiotics to prevent infection. Endoscopic mechanical lithotripsy with a single-use snare was performed in this case. However, due to the large size and hardness of the phytobezoar, the treatment effect was suboptimal, and based on the pertinent literature,^[1]^ she was orally administered sodium bicarbonate tablets (1 g/d) and Coca-Cola (500 mL/d) for 4 days. Follow-up gastroscopy after 4 days showed that the phytobezoar disappeared, and the symptoms significantly improved. The patient reported significant symptomatic relief throughout the treatment program, with outcomes exceeding expectations, she expressed great satisfaction with the treatment and completed the follow-up schedule as recommended. At the 14-day follow-up, the patient felt well and did not experience any symptoms.

## 3. Discussion

Bezoars are conglomerates of indigestible foreign materials found in the gastrointestinal tract; the phytobezoar of this case was an accumulation of indigestible fruit in the gastrointestinal tract. Gastric ulcers, gastrointestinal perforation, and intestinal obstruction were the main complications; intestinal obstruction caused by phytobezoars can result in severe complications. For example, a case report described a 45-year-old woman who developed symptoms such as abdominal colic, nausea, vomiting, abdominal distension, and intestinal obstruction after eating a large quantity of pears; she was ultimately diagnosed with small bowel obstruction caused by pear-induced phytobezoars.^[5]^ Acute pancreatitis triggered by causative agents, including alcohol consumption, gallstones, dyslipidemia, drugs, and infections, is frequently addressed. However, reports of acute pancreatitis caused by gastric bezoars are limited. Excessive consumption of persimmons, black dates, and other foods high in tannins is a dietary element that is closely linked to the development of phytobezoars.^[6–8]^ Mechanical injury of the phytobezoars to the stomach mucosa and the stimulation of gastric acid secretion may be the reasons why consuming many hawthorns in this instance was associated with the development of phytobezoar, acute pancreatitis, and gastric ulcers.^[9]^ This may have been caused by phytobezoar irritation of the gastrointestinal tract, which results in abnormal pancreatic fluid secretion and elevated pressure in the pancreatic ducts.^[10]^ While the causal relationship between phytobezoars and pancreatitis is plausible, it remains presumptive; further research is necessary to determine whether phytobezoars contributed factor in this patient. Phytobezoars can be managed through several treatment modalities, such as endoscopic intervention, chemical dissolution, and surgical procedures. Recent studies have investigated the efficacy of cola as a noninvasive treatment option, attributing its potential effectiveness primarily to the degradative properties of carbonic acid and phosphoric acid present in the beverage, which act on the fibrous components of the phytobezoars. A secure and efficient non-surgical therapeutic option for lithotripsy is oral sodium bicarbonate and carbonated beverages.^[11]^ The mechanism of carbonated beverages in the treatment of phytobezoars is mainly based on the interaction of their physicochemical properties with the intragastric environment; carbonated beverages are rich in carbon dioxide when entering the stomach. Carbon dioxide is rapidly released, which can increase the pressure in the stomach, prompting enhanced gastric peristalsis, which helps to loosen and disperse the phytobezoars. However, carbonated beverages are acidic and can neutralize the alkaline components of phytobezoars, and the primary constituents of plant phytobezoars, hemicellulose and pectin, can be hydrolyzed in an acidic environment, decreasing the hardness and adherence of the stones. Furthermore, the sugar in carbonated beverages may induce gastric hypertonicity, drawing water into the lumen and diluting gastric contents, thereby decreasing the structural integrity of phytobezoars. According to previous studies, phosphoric acid found in carbonated beverages can also react with the calcium ions in phytobezoars to create soluble calcium phosphates, which accelerates the dissolution of phytobezoars.^[12,13]^ It should be noted that this treatment is effective for phytobezoars, but endoscopic lithotripsy and other synergistic treatments are frequently needed for large, hard stones.^[14]^ Therefore, oral carbonate dissolution is a simple and effective non-surgical treatment for phytobezoars, and comprehensive therapeutic interventions, including dissolution of the phytobezoars, can successfully manage the inflammatory response and stabilize acute pancreatitis.^[8,9]^ The prolonged and excessive consumption of carbonated beverages may lead to various adverse health effects, particularly those related to metabolism. Such consumption can cause fluctuations in blood sugar levels, potentially triggering insulin resistance and increasing the risk of developing type 2 diabetes. Additionally, the high caloric content of these drinks is likely to contribute to weight gain and obesity. Carbonated beverages may also induce digestive discomfort, manifesting as increased gastric gas, abdominal distension, and belching. For individuals with gastroesophageal reflux disease, these drinks may exacerbate acid reflux symptoms. Furthermore, the imbalance between calcium intake and loss associated with carbonated beverage consumption may elevate the risk of osteoporosis, particularly in individuals with inadequate calcium intake. Populations advised to avoid carbonated drinks include those with diabetes, obesity, or weight management goals, as well as individuals with gastrointestinal disorders or osteoporosis. Although the application of cola in the treatment of phytobezoars has been extensively discussed, its efficacy, optimal dosage, treatment duration, and potential side effects require further validation through rigorous clinical research. This case underscores that a meticulous diagnostic workup, especially in the presence of multiple symptoms and abnormal findings, is crucial for determining the etiology and formulating a personalized, comprehensive treatment plan for patients with phytobezoar complicated by acute pancreatitis, ultimately aiming to enhance therapeutic efficacy and prognosis.

## 4. Conclusion

Through a thorough diagnosis and treatment of a 65-year-old female patient with phytobezoar and multimorbidity, we present a non-surgical treatment option after failed endoscopic procedures for phytobezoars in patients with multimorbidity. This approach helps avoid unnecessary surgeries in high-risk groups; it is simple, effective, and acceptable and is particularly beneficial in resource-limited settings. Although carbonated beverages proved effective in treating this particular case of phytobezoar, the efficacy of this method requires further validation through additional patient studies. This case illustrates the concepts and techniques of diagnosis and treatment for the coexistence of multiple pathologies. It also serves as a reference for the diagnosis and treatment of similar clinical cases.

## Author contributions

**Conceptualization:** Guobiao Luo.

**Data curation:** Guobiao Luo.

**Resources:** Guobiao Luo.

**Writing – original draft:** Guobiao Luo.

**Writing – review & editing:** Guobiao Luo.
